# Age-related cognitive decline in baboons: modeling the prodromal phase of Alzheimer's disease and related dementias

**DOI:** 10.18632/aging.103272

**Published:** 2020-05-19

**Authors:** Stephanny Lizarraga, Etienne W. Daadi, Gourav Roy-Choudhury, Marcel M. Daadi

**Affiliations:** 1 Southwest National Primate Research Center, Texas Biomedical Research Institute, University of Texas Health at San Antonio, San Antonio, TX 78227, USA; 2 Research Imaging Institute, Radiology, Cell Systems and Anatomy, University of Texas Health at San Antonio, San Antonio, TX 78227, USA

**Keywords:** aging, cognition, motor skills, CANTAB, actigraphy

## Abstract

The aging of brain cells and synaptic loss are the major underlying pathophysiological processes contributing to the progressive decline in cognitive functions and Alzheimer’s disease. The difference in cognitive performances observed between adult and aged subjects across species highlights the decline of brain systems with age. The inflection point in age-related cognitive decline is important for our understanding of the pathophysiology of neurodegenerative diseases and for timing therapeutic interventions. Humans and nonhuman primates share many similarities including age-dependent changes in gene expression and decline in neural and immune functions. Given these evolutionary conserved organ systems, complex human-like behavioral and age-dependent changes may be modeled and monitored longitudinally in nonhuman primates. We integrated three clinically relevant outcome measures to investigate the effect of age on cognition, motor function and diurnal activity in aged baboons. We provide evidence of a naturally-occurring age-dependent precipitous decline in movement planning, in learning novel tasks, in simple discrimination and in motivation. These results suggest that baboons aged ~20 years (equivalent to ~60 year old humans) may offer a relevant model for the prodromal phase of Alzheimer’s disease and related dementias to investigate mechanisms involved in the precipitous decline in cognitive functions and to develop early therapeutic interventions

## INTRODUCTION

Aging is currently an irreversible biological process characterized by a gradual deterioration in general health and function along side an increase in risk of disease and death [1]. In humans and nonhuman primates (NHP), aging is accompanied by both structural and physiological changes leading to breakdown in high-order brain systems [2, 3]. Imaging studies have demonstrated that aging leads to a reduction in brain size [4–6] due to shrinkage of the grey matter and to vascular-related changes in the white matter [7, 8]. The loss of neurons and changes in dendritic arborization, spines, and density of synapses have been attributed to grey matter shrinkage [9–12]; whereas degeneration or lesions due to ischemic injury are contributing factors to age-related changes in white matter [13, 14]. These changes to the brain are region specific. Previous studies have reported that the prefrontal cortex (PFC) is one the regions most affected by age, with significant decrease in synaptic density and cortical layer thickness [6, 15–19]. PFC plays an important role in working memory function, self-regulatory and goal-directed behaviors, all of which are vulnerable to aging [20]. Age-associated changes in PFC function can impair cognitive functions, learning and memory [20–26].

Early detection of age-associated cognitive dysfunction is crucial, as this provides a window of opportunity to understand the breakdown of brain systems and to implement interventions that may stop or limit the progression of diseases. The development of computer based testing tools along with traditional neuropsychological assessments has significantly improved the ability to detect age-associated changes in cognitive functions. Non-verbal based computerized tests, like CANTAB (Cambridge Neuropsychological Test Automated Battery), are reliable tests for identifying age-associated impairments in memory function [27–29]. CANTAB tasks have also been shown to be consistent for monitoring and assessing the cognitive functions in aged individuals [30, 31] and sensitive in identifying differences in working memory, attention and planning [5, 32, 33]. Among utilities for diagnostic measures, CANTAB has been recommended for mild cognitive impairment in Parkinson’s disease (PD) patients [34–36] and for detecting subtle cognitive impairments in sub-acute stroke [37, 38]. In NHP CANTAB was effective in identifying deficits in cognitive functions in various species including macaques [39, 40], baboons [41, 42] and marmosets [43, 44].

In the present study we addressed the question whether or not there is precipitous age-related cognitive and motor decline at a specific age. We integrated three clinically relevant outcome measures to address this question and investigate the effect of age on cognition, motor function and diurnal activity in adult and aged baboons. We used 1) CANTAB to identify age-associated differences in learning, shape discrimination and motivation; 2) The object retrieval task with barrier detour (ORTBD) to determine changes in motor function; and 3) Actigraphy analysis to investigate differences in baboons’ diurnal and nocturnal activities. We provide evidence of significant cognitive decline in aged baboons (~20 years old), including impairment of movement planning, learning novel tasks, simple discrimination and motivation.

## RESULTS

### Age associated differences in learning novel tasks in baboons (CANTAB touch task 1, TT1)

**Figure 1:**
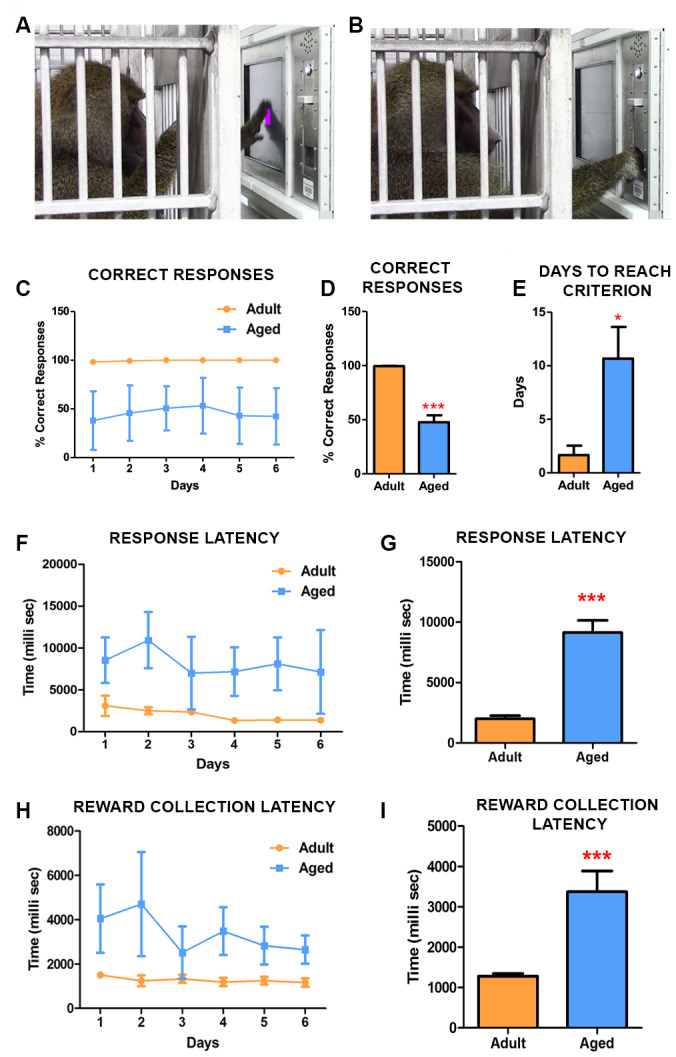
**CANTAB touch task 1 detects age-associated differences in learning novel tasks.** (**A**) Photograph depicting the baboon touching the stimulus displayed on the CANTAB screen. (**B**) Baboon retrieving the fruit pellet from the dispenser following a correct response on the task. (**C**) Daily performances demonstrated that adult baboons learned the task quicker and scored more correct responses than the aged ones. (**D**) The difference in performance in correct response was significantly different (Two-way ANOVA, ** p<0.05 Vs. adult). (**E**) The aged baboons took significantly more time to reach the criterion compared to the adult animals. The latencies to respond (**F** and **G**) and to collect pellets from the dispenser (**H** and **I**) were significantly slow in aged subjects.

The animals were split into 2 groups based on their ages: adult group (13±3 year old) and aged group (20±3 year old). To address the effect of age on the ability to discern and learn novel tasks, we tested baboons in CANTAB touch task I (TT1) with an unbaited screen (Figure 1A, 1B) (Video 1). The testing was performed until all animals reached the criterion (100% correct responses). In this simple task, the adult baboons reached criterion in the first day (Figure 1C) while in comparison the aged baboons clearly showed difficulty in learning the task and lagged behind with significant difference in performance (Figure 1C–1E). The response latency (Figure 1F, 1G) revealed that adult subjects exhibited a heightened attentional performance reflected by significantly better response accuracy and latency (Figure 1D, 1G). Furthermore, the latency to collect earned food pellets increased significantly in aged subjects indicating motivational or motor impairments (Figure 1H, 1I).

### Impairments in learning precision-based response tasks in aged baboons (CANTAB touch task 2, TT2)

**Figure 2:**
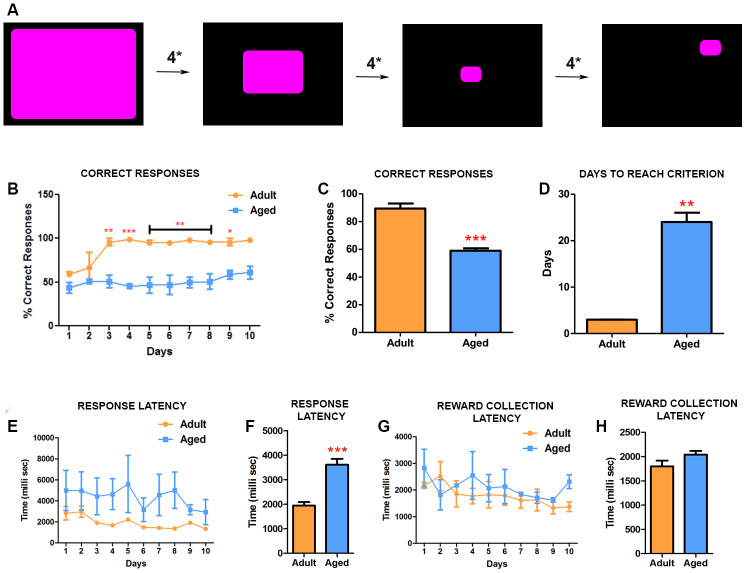
**CANTAB touch task 2 detects impairment in learning precision-based tasks in aged baboons.** (**A**) Illustration of CANATB touch task 2 showing the progressive decrease of the stimulus size that occurs every four consecutive successful responses (4*). After reaching final size the stimulus then appears at random locations on the screen. (**B**) Daily CANTAB TT2 task revealed that adult baboons performed significantly better than aged ones after 3 days (Two-way ANOVA, * p<0.05 Vs. adult). (**C**) The overall performance, measured by correct responses, of adult baboons was significantly better (*** p<0.001). (**D**) In comparison to aged baboons, adult baboons were significantly faster to reach criterion. (**E**) Quantitative analysis of the response latency between aged and adult baboons. (**F**) The latency to response is significantly longer in aged baboons compared to the adult subjects (** p<0.01). (**G** and **H**) The latency to collect rewards during the task was similar between the groups.

Before initiating this CANTAB task, all baboons were trained and reached criterion on the TT1. The TT2 comprised of two-tiered complexity. First, the baboons had to acclimatize to the operant condition of the stimulus shrinking in size (Figure 2A). The decrease in the size of the response area required the baboons to learn to touch the stimulus accurately to receive the reward. Once the stimulus reached its final size, it would then disappear and appear at a random location on the screen, which was the next level of complexity the baboons had to overcome. Optimal performance on this task requires integration of several cognitive processes, indexing attention, motivation and learning. Similarly to TT1, adult baboons learned the task faster, with high correct responses and fast response latencies. By day 3 adult subjects reached the criterion (90% correct responses) for the test and consistently maintained this performance thereafter (Figure 2B) while the aged baboons had difficulty in learning the task. The number of correct responses in each session was consistently fewer in aged baboons indicating low response accuracy (Figure 2B, 2C). Despite continuing the testing for 26 days the aged baboons never reached criterion for the test and were significantly different from adults (Figure 2D) (P=0.0001). Similarly to TT1, the aged baboons exhibited a significant (P=0.0007) increased latency in responding to the stimulus compared to adult baboons (Figure 2E, 2F), while no significant differences in reward collection latency was observed (Figure 2G, 2H). Together, these data suggests that aged baboons exhibit deficiencies in attention, learning and memory.

### Effects of age on simple discrimination (SD)

**Figure 3:**
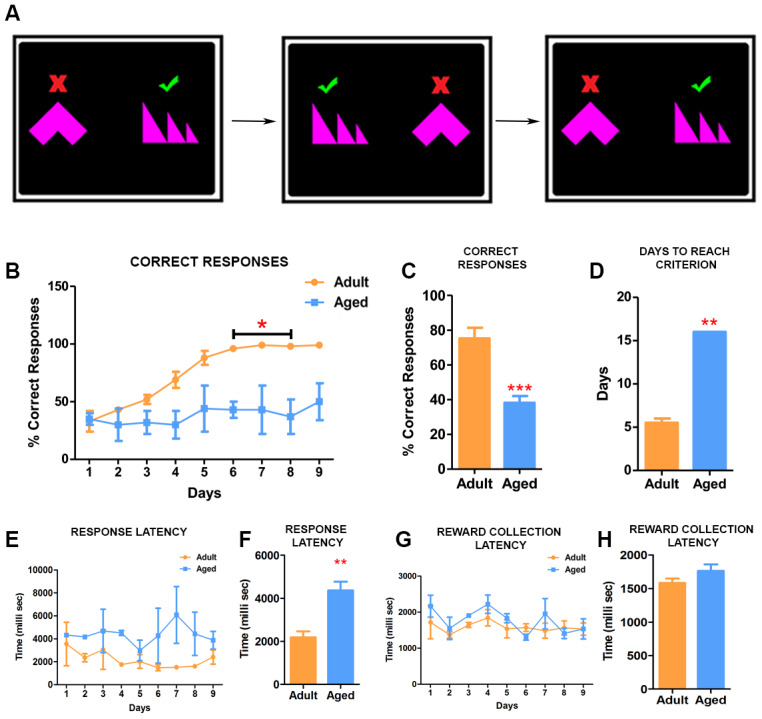
**Simple discrimination task reveal deficits in learning.** (**A**) Illustration depicting the set up for the simple discrimination task. One shape (green check mark) is associated with delivery of reward during the task. (**B**) Daily performances of simple discrimination task showed that adult baboons learned to identify the correct shape associated with rewards and performed consistently better than aged baboons. The difference in performance on the task from day 6 was statistically significant (Two-way ANOVA, * p<0.05). (**C**) The adult baboons overall performed significantly better compared to the aged baboons on the task (*** p<0.001). (**D**) The aged baboons took longer to reach criterion (** p<0.01). (**E**) Graph depicting the response latency of adult and aged baboons during each day of the task. (**F**) The latency to respond was significantly longer in aged baboons compared to the adult subjects (** p<0.01). (**H** and **G**) Quantitative analysis of pellet collection latency between adult and aged baboons showing no significant differences.

We next investigated the effect of age on the ability to learn to discriminate between shapes (Figure 3A). For SD, the baboons had to learn by trial and error to identify the right shape associated with fruit pellets and ignore the shape that does not provide a reward (Figure 3A) (Video 2). Adult baboons showed a steady and rapid improvement in their accuracy on the task (Figure 3B). They were able to learn to associate the right shape with reward and reached criterion by day 6 of the testing (Figure 3B). In contrast, the aged baboons did not show improvement over time and they exhibited difficulties to associate the correct shape with the reward by continuing to touch indiscriminately both shapes resulting in a significantly lower number of correct responses (Figure 3C). Further continuation of the testing showed that aged baboons reached criterion for the test on day 16, a significantly longer time span than adults (Figure 3D). In addition, and similarly to the TT2 task, aged baboons showed significant increase in latency to respond to the stimulus (Figure 3E, 3F) with no significant differences in reward collection time (Figure 3G, 3H).

### Age-dependent deficits in motivation

**Figure 4:**
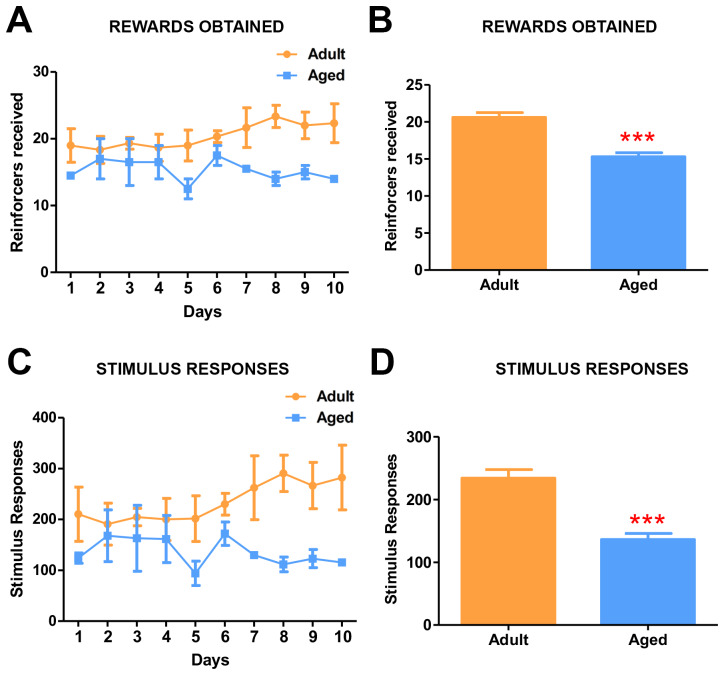
**Progressive ratio task detects the effects of age on motivation.** (**A**) Graph depicting the number of rewards obtained by the adult and aged baboons during each day of the progressive ratio task. (**B**) Quantitative analysis of rewards obtained during progressive task. Adult baboons obtained significantly more rewards compared to the aged group (*** p<0.001). (**C**) Graph depicting the number of responses made by the baboons during progressive ratio task. (**D**) Quantitative analysis of stimulus responses on the progressive task. Aged baboons made significantly fewer response touches compared to adult baboons (*** p<0.001).

The effect of age on the motivation to collect the reward was assessed using the progressive ratio (PR) schedule of reinforcement. The task required the baboons to touch the stimulus by increments of 1 (1,2,3,4, 5 and so on), increasing the number of responses to continue to earn the reward (reinforcer). The subjects showed a marked age-related difference in the effort they were willing to expend in pursuit of the reward. Adult baboons continued to touch the screen longer to obtain the rewards and reached breaking point after earning a maximum of 27 fruit pellets (Average= 20.5 rewards obtained/day), which required 27 consecutive touches on the screen (Figure 4A, 4B). In contrast, the aged baboons showed a significantly low breaking point of 16 (average =14.5 rewards obtained/day) (Figure 4A, B) (Video 3). The stimulus-responses to rewards learning data (Figure 4C, 4D) confirmed the breaking points for both groups.

### Age-associated differences in motor function in baboons

**Figure 5:**
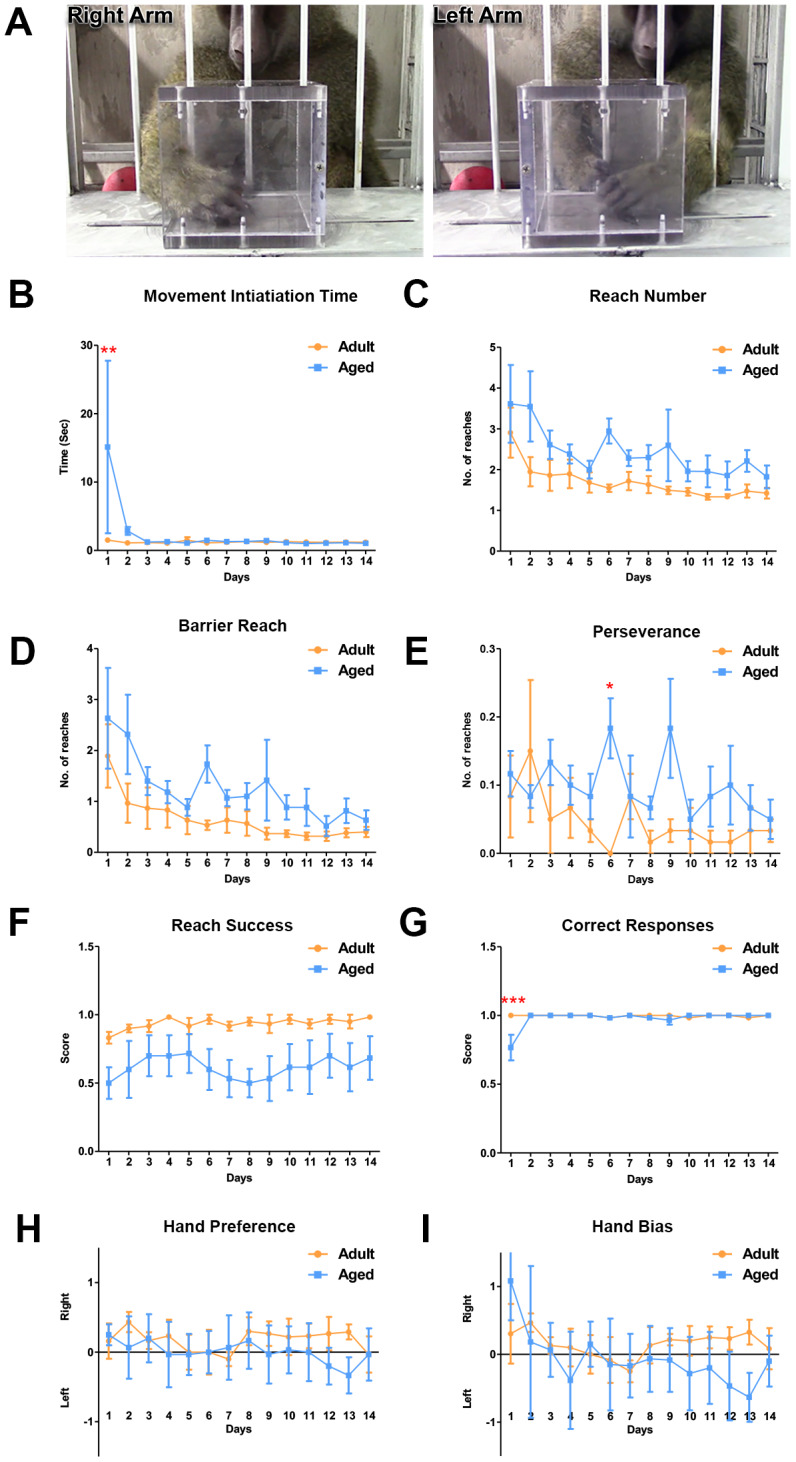
**Differences in motor function between adult and aged baboons on the object retrieval task with barrier detour.** (**A**) Representative images of the baboon performing the object retrieval task. The baboons had to learn to by-pass the transparent barrier and reach for the reward through the open side of the box. (**B**) On day 1, aged baboons took significantly longer to initiate a response after the screen was raised compared to adult baboons (Two-way ANOVA, ** p<0.01). No significant differences were seen in (**C**). Reach number. (**D**) Barrier reach. (**E**) Perseverance (except day 6) and (**F**) Success. (**G**) Aged baboons committed significantly more errors on day 1 of the task compared to adult baboons (Two-way ANOVA, *** p<0.001). No hand preference (**H**) or hand bias (**I**) was observed.

The object retrieval task with barrier detour (ORTBD) was used to measure the motor and cognitive functions of the adult versus aged baboons. During the task, reaching and retrieving the reward measured motor function while cognition was measured by their ability to learn to bypass the barrier when the orientation of the opening of the transparent box was altered [45, 46]. The baboons received no prior training for the test. On day 1, the aged baboons required a significantly longer time to make an attempt to retrieve the reward (movement initiation time) once the screen was raised (Figure 5B). In addition, the aged baboons were less competent on the task during the first day compared to the adult baboons as seen in the low number of successfully retrieved rewards (Figure 5G). The baboons learned to retrieve the reward by circumventing the barrier, indicated by the gradual decrease in the reach number and barrier touches and no significant differences were seen between the groups (Figure 5C). Although not significant, adult subjects performed better on the barrier reach and reach success (Figure 5D, 5F). The perseverance measures showed significance on day 6 suggesting an inhibitory deficit. The task offered an equal number of opportunities to use either the right or left arm to retrieve the reward during the trials. The hand bias and hand preference were close to zero for both adult and aged baboons (Figure 5H, 5I).

### Activity analysis of the baboons

**Figure 6:**
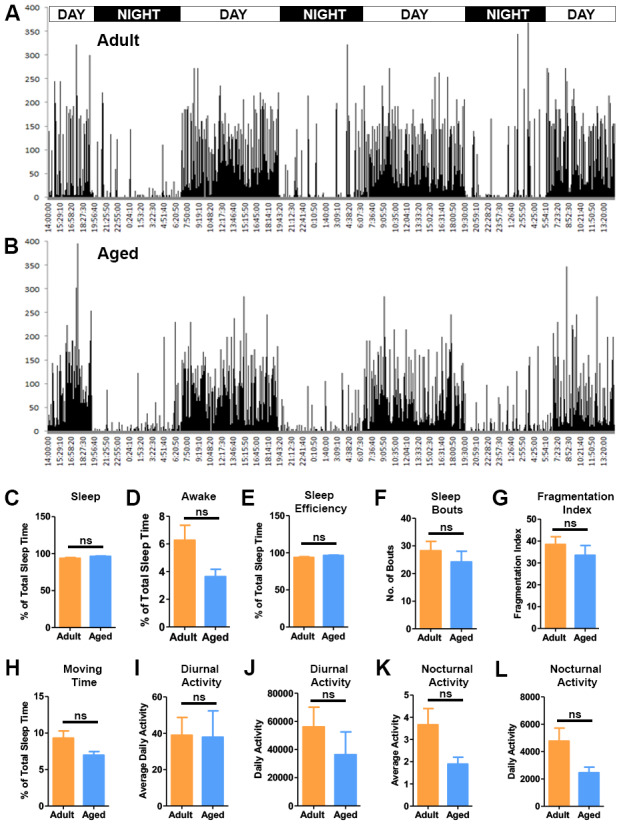
**Activity analysis of the baboons.** Representative actograms of the adult (**A**) and aged baboons (**B**) from the actiwatch analysis. The actograms demonstrate the activity of baboons during a period of 72 hrs. Sleep quality parameters were quantified from the night-time activity analysis. (**C**) Percent sleep time, (**D**) Percent awake time, (**E**) Sleep efficiency, (**F**) Sleep bouts and (**G**) Fragmentation index. No significant differences were observed between the two groups in sleep quality parameters. The actogram data was also used to quantify movement parameters like (**H**). Time spent moving during sleep, (**I**) Average nocturnal activity, (**J**) Daily nocturnal activity, (**K**) Average diurnal (day time) activity and (**L**) Daily diurnal (day time) activity. No significant (ns) differences were seen between the groups in the movement parameters.

The daily activity of the baboons was recorded using the actiwatch mini (Figure 6A, 6B). The actiwatches were placed in the baboons’ collars and the activity data was recorded for a period of 72 hrs then analyzed using the sleep analysis-7 software [46]. The actograms demonstrated the diurnal activity pattern in the baboons that corresponded with the light-dark cycle (lights on 7AM - lights off 7PM) of the housing (Figure 6A, 6B). In the morning between 7:00 and 8:00 AM the actograms depicted a sudden increase after a long period of quiescence indicating the baboons waking up. The activity then gradually peaked until noon and continued into the evening (Figure 6A, 6B). Analysis of the actiwatch data indicated more daytime activity in the adult baboons. The activity gradually decreased once the lights went off. During night actograms showed prolonged periods of inactivity interspersed with many small peaks of activity attributed to movements during sleep (Figure 6A, 6B). Next we investigated the quality of sleep among the baboons by comparing different sleep fragmentation parameters (Figure 6C–6L). The total sleep time, sleep efficiency and number of wake bouts was similar between the groups with no statistical differences (Figure 6C, 6E, 6F).

## DISCUSSION

NHP models have played a vital role in aging research as they manifest many of the structural and physiological modifications in the brain linked to chronological aging. In addition, NHPs visual orientation and ability to perform complex behavioral tasks are well suited to investigate the effect of age on different cognitive domains [47, 48]. In this study, we investigated cognitive and motor performances and sleep patterns in adult (13±3 year old) and aged (20±3 year old) baboons. We report a significant decline in cognition between adult and aged baboons. CANTAB results demonstrated an effect of age on learning a novel task, a precision-based task and a simple discrimination task. The results from the PR task indicated an effect of age on motivation. The ORTDB demonstrated age-related impairment in performing novel tasks, whereby a longer initiation time and fewer successful attempts were observed. The data suggests that ~20 years of age in baboons is a critical period during which there is a significant decline in cognition.

Although changes in cognition as a function of age has been well documented [49–55], most studies focus on young versus adult/aged subjects and rarely address the differences between adult and aged subgroups or the specific inflection point where a precipitous decline in cognitive abilities takes place. It has been demonstrated that crystalized cognitive abilities, the skills and knowledge acquired over an individual’s lifetime, are decreased to a lesser extent by aging in comparison to fluid cognitive functions, such as processing speed, working memory, attention and executive control. Fluid cognitive functions show a gradual decline with age and can be evaluated by measuring a subject’s ability to process and use new information in problem solving (reviewed in [56]). In our study, we investigated the ability of adult and aged baboons to learn a novel task without any pre-training. Unlike previous studies that have used food as a bait to train monkeys to touch the screen [41, 42, 57], we adapted the unbaited approach to measure the baboons’ ability to solve problems and learn novel tasks. The baboons only received reinforcement familiarization prior to the testing thus the monkeys had to learn by trial and error to touch the response cue to obtain the rewards (TT1). Similarly in the precision-based response task (TT2), the baboons had to adapt to the shrinking response cue to get the fruit pellets. These paradigms presented the baboons with novel conditions prompting them to learn to use new information in problem solving.

In human subjects, memory and neuroplasticity for learning new information decreases with age. Assessment of “new learning” using the method of loci (MoL) approach demonstrated that young adults had better episodic memory when compared to old individuals [58]. Similarly our evidence suggests that the adult baboons excelled in performance on both paradigms of learning new tasks. They were quick to learn the relationship between touching the stimulus cue and receiving reward and performed significantly better compared to the aged baboons. In the SD task that measures associative learning, the aged baboons demonstrated significant deficits in associating the right shape with reward and made significantly more errors. In addition the aged baboons required an extended period on CANTAB to reach criterion set for SD. The latency to respond during the stimuli presentation was significantly longer in the aged subjects. This suggests that aged baboons exhibit attentional decline, their decision-making is impaired or this may be due to age-related slowness in movement execution. However, after the first day on the ORTBD task aged baboons performed similarly to adults on movement initiation time and correct response, suggesting that slowness in task performances may be due to cognitive deficits. Alterations in medial prefrontal cortex and nucleus accumbens circuitry have been reported as involved in the mechanism behind difficulties seen in decision-making in older individuals [54, 59–62]. However, further studies are needed to demonstrate age-related alterations in the executive functions and its neural substrate in baboons.

The PR task measures motivation of subjects to work for the reinforcers with minimum cognitive load and has been widely used in both humans and experimental animals [63–65]. Our results of the PR task demonstrated that there is an age-dependent decrease in the level of motivation in baboons. The adult baboons were more motivated as they made significantly more touch responses and received more reinforcers than the aged baboons. Interestingly in both groups performance on the PR task remained consistent during the course of testing with the aged baboons performing less than the adults. These data are consistent with previous reports showing that NHPs quickly achieve stability on motivational tasks and their performance remains consistent over long periods of time [43, 57]. The mesolimbic circuit of the brain is a major neural substrate of behaviors associated with motivation, reinforcement learning, and reward-associated responses. Compelling evidence suggests the dopaminergic system that projects from the ventral tegmental area (VTA) to the nucleus accumbens (NAcc) and other structures of the forebrain is a major substrate of reward and reinforcement [66, 67]. Interestingly, a recent study demonstrated an age-related decrease in VTA–NAcc coupling in the context of motivation level [68]. However, further studies are required to demonstrate the implication of the dopaminergic system in the declined motivation seen in aged baboons.

In addition to CANTAB analysis, we used the ORTBD task to evaluate both motor and cognitive functions in baboons. During testing the baboons had to modify their reaching strategy and learn how to bypass the transparent barrier. The task required complex succession of motor and cognitive planning. Similar to the results of CANTAB, when first exposed to the ORTBD the baboons exhibited significant differences and age-dependent performances in movement planning, initiation and number of correct responses during the learning phase. On the first day of testing the adult baboons performed better and exhibited a significantly shorter latency to reach for the reward (initiation time) and made significantly more correct attempts compared to the aged baboons. These deficits did not appear to be related to motor skills since the scores of motor problems, reach success and reach number were not significantly different between the two groups, while the correct response was significantly different during the initial day of learning. Together these data suggest the increase in initiation time observed in aged baboons may be due to an impaired cognitive response during the planning strategy. The ORTBD task involves altering the spatial projection of movement and planning the trajectory of execution to reach around the transparent barrier and retrieve the reward. This task engages the prefrontal and motor cortices and the striatum, all of which undergo significant synaptic alterations and cell loss during aging [19–26] and neurodegenerative diseases [46, 69–72].

Differences in cognition and motor skills between young and old subjects have been well studied in animal models and human subjects [50–53]; however, studies investigating the differences in cognitive function among adult and elderly subjects are rare and the age at which cognitive and motor skills begin to decline is still not clear. Studies on postmortem human brains have suggested an increase in accumulation of oxidative damage in the brain with aging, with 60 years as the potential break point [73]. These findings are consistent with our results and suggest that approximately 20 years of age in baboons (equivalent to approximately 60 human years [74]) is a period of precipitous decline in brain systems function and in particular cognition. However, further studies involving a larger number of baboons of both genders to investigate the development of beta-amyloid plaques [75], hyperphosphorylated tau tangles [76] and synapse disruptions with age in brain regions, such as the PFC and hippocampus are necessary next steps for the characterization of this model. Nevertheless, this naturally-occurring age-related precipitous decline in cognitive functions in baboons may offer a relevant experimental model to understand the etiology and to develop early interventions during the prodromal phase of AD and related dementias.

## MATERIALS AND METHODS

### Animals

All experiments were performed on Olive baboons (*Papio anubis*) from the Southwest Nonhuman Primate Research Center (SNPRC) colony. The behavioral procedures were performed in strict accordance with the recommendations proposed in the Guide for the Care and Use of Laboratory Animals, National Research Council U. S. A. Six healthy baboons were studied: three adult baboons 13±3 year old (N=3, 2 males, 1 female) and three aged baboons 20±3 year old (N=3 females). The animals weighed between 21-30 kilograms (within normal baboon body weight for their respective age and sex group). All animals underwent a health screen, including computerized tomography (CT) and magnetic resonance imaging (MRI) and showed no neurological abnormalities. The study protocol was approved by the Institutional Animal Care and Use Committee of Texas Biomedical Research Institute. All NHP held and used within the SNPRC program of care at the Texas Biomedical Research Institute are maintained under conditions that meet the USDA Animal Welfare Regulations, OLAW standards, National Institute of Health (NIH) guidelines, as stated in the Guide for the Care and Use of Laboratory Animals (81h Edition, 2010), NAS-ILAR recommendations, and AAALAC accreditation standards for these species. Texas Biomed, including the SNPRC as a component of its overall program, is fully accredited by AAALAC International. The center promotes social housing caging, with structural complexities for environmental enrichment with detailed observation of ongoing animal activities. Inside the animal quarters, the temperature and humidity is maintained suitable for baboons, at 80°F and 60% respectively. Animals are fed constant nutrition, complete life-cycle commercial monkey chow, supplemented daily with fruits and vegetables. Municipal drinking water is available to animals at all times. All research activity has been conducted in accordance with the IACUC oversight process. The SNPRC employs a large number of full-time professional staff members to provide expertise in program administration, animal husbandry, clinical medicine, psychological well-being, facilities maintenance, animal records, and technical research support. This study has no procedures or euthanasia of animals thus discomfort, distress or pain are not applicable. All animals are enrolled in the environmental enrichment program. Enrichment provided to the animals consists of social contact, structural enrichment (e.g., perches, swings), manipulable enrichment (e.g., chew toys, balls), nutritional enrichment (e.g., fruit, grain), sensory enrichment (e.g., television, radio), and occupational enrichment (e.g., food puzzles). All enrichment provided is documented, and any deficiencies are addressed. Although not performed in this study, the veterinarians at the SNPRC perform humane euthanasia of animals and in accordance with the professional principles and practices specified by the American Veterinary Medical Association Guidelines for the Euthanasia of Animals: 2013 Edition. Animals destined for euthanasia are injected intraperitoneally with lethal dose of sodium pentobarbital (100 mg/Kg) followed by transcardiac perfusion with phosphate buffered saline and 4% paraformaldehyde for tissue processing.

### Cambridge neuropsychological test automated battery (CANTAB)

Nonhuman primate CANTAB intellistation (Model 80950A, Lafayette instruments) was used in the current study. The intellistation housed an onboard computer, touch screen monitor (approximately 15 inch response area) and pellet reward dispenser enclosed in a heavy-duty metal frame. The CANTAB system was mounted on a movable stainless steel cart, for easy of transportation between cages. CANTAB and Whisker Server software provided with the intellistation was used to generate different visual stimuli, audio cues, as well as for collection and storage of data. Fruit crunchies (F05798, Bio-serv), consisting of a medley of fruit flavored (banana, apple, orange and grape) pellets were used for rewards. The night before (7:00 PM) testing, food was removed from the cages and water remained available. Testing was conducted between 10:00 AM-11:00AM on weekdays. The baboons were fed 30 minutes after the end of each session during which they received a large variety of fruits, as enrichment, in addition to the regular NHP chow. For reinforcement familiarization CANTAB intellistation was placed in front of the animal’s cage and a reward tone (1000 Hz; 1 second) was played before release of the fruit pellets from the dispenser. This was done for 2 sessions for each animal.

### CANTAB touch training test I (TT1)

The touch screen displayed a single purple (Red=255; Green=0; Blue=255) full screen stimulus (1000 notional units wide and 720 units high) and a response anywhere on the stimulus rewarded the subjects. The stimulus display was coupled with a link sound (100Hz, 0.1 second). The maximum number of trials was 50 and maximum time for each session was 30 minutes. The inter-trial interval was 5 seconds and each time the stimulus was preceded by a standard tone. Correct responses were rewarded with a fruit pellet coupled with the reward tone (1000 Hz; 1 second) whereas missing the stimulus or an incorrect responses was accompanied by a buzzer tone (40-Hz; 0.2 sec) without a fruit pellet. During testing the CANTAB apparatus was placed in front of the baboon’s modified cage. The baboons did not receive any prior training and no additional cues (visual or food) were provided during the test.

### CANTAB touch training test II (TT2)

The testing parameters were same as TT1: maximum number of trials was 50 and maximum time for each session was 30 minutes. The test started with the touch screen displaying a full screen stimulus (purple square of 1000 notional units wide and 720 units high). Following every 4 consecutive correct touches the stimulus was shrunk to sizes 800X675, followed by 600X600, 480X480, 360X360, 240X240 to a final size of 120X120. Once the stimulus size reached its final size (120X120) the stimulus disappeared and appeared at random locations on the screen. Correct responses were rewarded with a fruit pellet coupled with the reward tone. Incorrect responses were not rewarded and were accompanied by buzzer tone. The testing was done 5 days a week and continued until animal reached criterion i.e. 90% correct responses (45 out of 50 trials) in each session.

### CANTAB simple discrimination (SD)

The CANTAB monitor displayed two purple colored stimuli that differed in shape (univcam_IDED_Shape 12 and 43, from CANTAB library) on the left and right side of the screen (locations: 250,75; 750,375). Touching only one of the stimuli (correct choice) resulted in delivery of the fruit pellet. The stimuli switched location randomly and touching the wrong stimulus shape resulted in buzzer tone without any reward. Following the correct response, the shape assigned correctly remained on the screen during the delivery of reward. The length of each session was 30 minutes and the total number of trials in each session was 60. Inter-trial interval was 5 seconds. Testing was done daily and continued until the animals reached criterion (90% correct responses, 54 out of 60 trials).

### CANTAB progressive ratio (PR) task

A single stimulus (univcam _IDED_Shape 8 from CANTAB library) was displayed at the center of the screen (Location: 500,375). The number of touches to receive the reinforcer (fruit pellet) started with one and gradually increased by an increment of 1. Successful responses were marked both aurally (1kHz tone) and visually (color change from purple to white). The maximum length of each testing session was 30 minutes and testing was stopped if the baboon became non-responsive for a 3-minute period (breaking point).

### Object retrieval task with barrier detour (ORTBD)

Object retrieval task with barrier detour was used to evaluate motor and cognitive function in the baboons as previously described [45, 46]. Briefly, object retrieval with a barrier detour is a reward based behavioral testing system used for assessing motor and cognitive functions in NHP. The task requires the test subject to retrieve a reward (grapes) from the open side (bypassing the barrier) of a transparent box, which is fastened to a tray welded on to the cage. For the current study, the testing apparatus was modified to fit the baboons’ cages. Behavioral analysis was done for 14 days with 20 trials per session. During each trial, the orientation of the open side of the box was randomly changed to the left, right or facing towards the animal. The entire process was recorded using a video camera and the recordings were later used for scoring and data analysis. During each trial, the following responses were scored (1) ability of the animal to reach the front, left, or right side of the box, scored under the term “reach act”; (2) hand of choice for the reach, either left or right, scored under the term “hand used”; (3) the outcome of the reach, either success or failure, scored under result section.

Using the above parameters, we analyzed additional variables; 1) Reaching disability (Motor problem): Reaching into the open side of the box but without retrieving the reward. 2) Movement initiation: Latency from the screen being raised to the subject touching the box or reward. 3) Execution: Retrieving the reward from the box on the first reach of the trial (indicates competence on the task). 4) Correct: Eventually retrieving the reward from the box on the trial (>1 reach on the trial to retrieve the reward as unlimited reaches per trial were allowed). 5) Reach number: Number of times the animal made an attempt and touched the box. 6) Hand preference: Hand (left or right) that the subject used for the first reach of the trial. 7) Hand bias: Total number of left and right hand reaches on each trial. 8) Awkward reach: Reaching with the hand farthest away from the box opening (either the left or right side). 9) Perseverative response: Repeating a reach to the side of the box that was previously open but then closed. 10) Barrier reach: Reaching and touching the closed side of the test box. The results from the data analysis were plotted using Graph pad prism statistical software.

### Actigraphy analysis

The diurnal behavior of the baboons was monitored using the actiwatch mini (Cam*n*tech, UK). The actiwatches were placed on collars that were custom made using 1 ½” natural polyester tape. Care was taken to ensure that the collars did not restrict the animal’s respiration, feeding or daily activity. The baboons were first trained and acclimatized to put the collars on for 3 days and then the actiwatches were placed and activity was recorded for 72 hrs. Following the IACUC and our standard operating procedures for short-term (10-30 min) veterinary care, treatment or collar placement on baboons, we used low dose anesthesia: ketamine (5-10 mg/kg, IM). The data from the actiwatches was downloaded using the actiwatch activity and sleep analysis-7 software (Cam*n*tech, UK). For sleep analysis, the period of sustained quiescence during the dark phase (7:00 PM -7:00 AM) was analyzed. The sleep analysis-7 software was used to quantify the sleep quality and wakeful periods. The duration of sleep time was corrected for individual variations in animals to fall asleep at different times of the evening; thus, we kept the period of sleep time analyzed the same for all of the animals. For daytime activity analysis, the movement of animals during the light phase at a resolution of every minute was generated using the actiwatch software. The data was then exported to excel and plotted using the Graph pad prism statistical software.

### Nonparametric circadian rhythm analysis (NPCRA)

NPCRA was performed on the Actiwatch data using the sleep analysis-7 software. The following established nonparametric indices of rhythmicity were generated: IS (Inter-daily stability): degree of regularity in the activity-rest pattern of the animal during a 24hr cycle. IV (Intra-Daily variability): degree of fragmentation of activity-rest periods. L5 (Lowest activity): average activity level for the least active five hours. M10 (Maximal activity): average activity level for the most active ten hours. Onset of L5 and M10 indicates the average time of the start of the least active 5-hour period (L5) and the most active 10-hour period (M10) during a circadian cycle and denotes the degree of coordination of individual’s circadian cycle with a normal 24-h cycle. Relative amplitude is estimated by dividing the difference between M10 and L5 period with the sum of M10 and L5. Relative amplitude has a range between 0-1 and higher values indicate a rhythm with higher amplitude. The data was then exported to excel and plotted using the Graph pad prism statistical software.

### Statistics

Statistical analysis was done with Graph Pad Prism statistical software. Significance in differences between 2 groups was performed by applying Student’s t-test where appropriate. For comparison of multiple groups, Two-Way ANOVA with Bonferroni post-hoc analysis was performed to identify the significant differences. A P-value of less than 0.05 was considered to be statistically significant.

## Supplementary Material

Supplementary Video 1

Supplementary Video 2

Supplementary Video 3

## References

[1] Hayflick L. How and why we age. Exp Gerontol. 1998; 33:639–53. 10.1016/s0531-5565(98)00023-0 9951612

[2] Buckner RL. Memory and executive function in aging and AD: multiple factors that cause decline and reserve factors that compensate. Neuron. 2004; 44:195–208. 10.1016/j.neuron.2004.09.006 15450170

[3] Bishop NA, Lu T, Yankner BA. Neural mechanisms of ageing and cognitive decline. Nature. 2010; 464:529–35. 10.1038/nature08983 20336135 PMC2927852

[4] Resnick SM, Pham DL, Kraut MA, Zonderman AB, Davatzikos C. Longitudinal magnetic resonance imaging studies of older adults: a shrinking brain. J Neurosci. 2003; 23:3295–301. 10.1523/JNEUROSCI.23-08-03295.2003 12716936 PMC6742337

[5] Raz N, Ghisletta P, Rodrigue KM, Kennedy KM, Lindenberger U. Trajectories of brain aging in middle-aged and older adults: regional and individual differences. Neuroimage. 2010; 51:501–11. 10.1016/j.neuroimage.2010.03.020 20298790 PMC2879584

[6] Fotenos AF, Snyder AZ, Girton LE, Morris JC, Buckner RL. Normative estimates of cross-sectional and longitudinal brain volume decline in aging and AD. Neurology. 2005; 64:1032–39. 10.1212/01.WNL.0000154530.72969.11 15781822

[7] Lindemer ER, Greve DN, Fischl BR, Augustinack JC, Salat DH. Regional staging of white matter signal abnormalities in aging and alzheimer’s disease. Neuroimage Clin. 2017; 14:156–65. 10.1016/j.nicl.2017.01.022 28180074 PMC5279704

[8] Safaiyan S, Kannaiyan N, Snaidero N, Brioschi S, Biber K, Yona S, Edinger AL, Jung S, Rossner MJ, Simons M. Age-related myelin degradation burdens the clearance function of microglia during aging. Nat Neurosci. 2016; 19:995–98. 10.1038/nn.4325 27294511 PMC7116794

[9] Kemmotsu N, Girard HM, Kucukboyaci NE, McEvoy LK, Hagler DJ Jr, Dale AM, Halgren E, McDonald CR. Age-related changes in the neurophysiology of language in adults: relationship to regional cortical thinning and white matter microstructure. J Neurosci. 2012; 32:12204–13. 10.1523/JNEUROSCI.0136-12.2012 22933802 PMC3475615

[10] Bonasera SJ, Arikkath J, Boska MD, Chaudoin TR, DeKorver NW, Goulding EH, Hoke TA, Mojtahedzedah V, Reyelts CD, Sajja B, Schenk AK, Tecott LH, Volden TA. Age-related changes in cerebellar and hypothalamic function accompany non-microglial immune gene expression, altered synapse organization, and excitatory amino acid neurotransmission deficits. Aging (Albany NY). 2016; 8:2153–81. 10.18632/aging.101040 27689748 PMC5076456

[11] Giorgio A, Santelli L, Tomassini V, Bosnell R, Smith S, De Stefano N, Johansen-Berg H. Age-related changes in grey and white matter structure throughout adulthood. Neuroimage. 2010; 51:943–51. 10.1016/j.neuroimage.2010.03.004 20211265 PMC2896477

[12] Scahill RI, Frost C, Jenkins R, Whitwell JL, Rossor MN, Fox NC. A longitudinal study of brain volume changes in normal aging using serial registered magnetic resonance imaging. Arch Neurol. 2003; 60:989–94. 10.1001/archneur.60.7.989 12873856

[13] Dumitriu D, Hao J, Hara Y, Kaufmann J, Janssen WG, Lou W, Rapp PR, Morrison JH. Selective changes in thin spine density and morphology in monkey prefrontal cortex correlate with aging-related cognitive impairment. J Neurosci. 2010; 30:7507–15. 10.1523/JNEUROSCI.6410-09.2010 20519525 PMC2892969

[14] Tullberg M, Fletcher E, DeCarli C, Mungas D, Reed BR, Harvey DJ, Weiner MW, Chui HC, Jagust WJ. White matter lesions impair frontal lobe function regardless of their location. Neurology. 2004; 63:246–53. 10.1212/01.wnl.0000130530.55104.b5 15277616 PMC1893004

[15] Peters R. Ageing and the brain. Postgrad Med J. 2006; 82:84–88. 10.1136/pgmj.2005.036665 16461469 PMC2596698

[16] Raz N, Lindenberger U, Rodrigue KM, Kennedy KM, Head D, Williamson A, Dahle C, Gerstorf D, Acker JD. Regional brain changes in aging healthy adults: general trends, individual differences and modifiers. Cereb Cortex. 2005; 15:1676–89. 10.1093/cercor/bhi044 15703252

[17] Goldman-Rakic PS, Brown RM. Regional changes of monoamines in cerebral cortex and subcortical structures of aging rhesus monkeys. Neuroscience. 1981; 6:177–87. 10.1016/0306-4522(81)90053-1 6111765

[18] Beckman D, Ott S, Donis-Cox K, Janssen WG, Bliss-Moreau E, Rudebeck PH, Baxter MG, Morrison JH. Oligomeric Aβ in the monkey brain impacts synaptic integrity and induces accelerated cortical aging. Proc Natl Acad Sci USA. 2019; 116:26239–46. [Epub ahead of print]. 10.1073/pnas.1902301116 31871145 PMC6936351

[19] Peters A, Sethares C, Moss MB. The effects of aging on layer 1 in area 46 of prefrontal cortex in the rhesus monkey. Cereb Cortex. 1998; 8:671–84. 10.1093/cercor/8.8.671 9863695

[20] Morrison JH, Baxter MG. The ageing cortical synapse: hallmarks and implications for cognitive decline. Nat Rev Neurosci. 2012; 13:240–50. 10.1038/nrn3200 22395804 PMC3592200

[21] Rubin LH, Meyer VJ, J Conant R, Sundermann EE, Wu M, Weber KM, Cohen MH, Little DM, Maki PM. Prefrontal cortical volume loss is associated with stress-related deficits in verbal learning and memory in HIV-infected women. Neurobiol Dis. 2016; 92:166–74. 10.1016/j.nbd.2015.09.010 26408051 PMC4808495

[22] Bloss EB, Janssen WG, Ohm DT, Yuk FJ, Wadsworth S, Saardi KM, McEwen BS, Morrison JH. Evidence for reduced experience-dependent dendritic spine plasticity in the aging prefrontal cortex. J Neurosci. 2011; 31:7831–39. 10.1523/JNEUROSCI.0839-11.2011 21613496 PMC3398699

[23] Rapp PR, Amaral DG. Evidence for task-dependent memory dysfunction in the aged monkey. J Neurosci. 1989; 9:3568–76. 10.1523/JNEUROSCI.09-10-03568.1989 2795141 PMC6569903

[24] Herndon JG, Moss MB, Rosene DL, Killiany RJ. Patterns of cognitive decline in aged rhesus monkeys. Behav Brain Res. 1997; 87:25–34. 10.1016/s0166-4328(96)02256-5 9331471

[25] Arnsten AF, Cai JX, Steere JC, Goldman-Rakic PS. Dopamine D2 receptor mechanisms contribute to age-related cognitive decline: the effects of quinpirole on memory and motor performance in monkeys. J Neurosci. 1995; 15:3429–39. 10.1523/JNEUROSCI.15-05-03429.1995 7751922 PMC6578230

[26] Moore TL, Schettler SP, Killiany RJ, Herndon JG, Luebke JI, Moss MB, Rosene DL. Cognitive impairment in aged rhesus monkeys associated with monoamine receptors in the prefrontal cortex. Behav Brain Res. 2005; 160:208–21. 10.1016/j.bbr.2004.12.003 15863218

[27] Robbins TW, James M, Owen AM, Sahakian BJ, Lawrence AD, McInnes L, Rabbitt PM. A study of performance on tests from the CANTAB battery sensitive to frontal lobe dysfunction in a large sample of normal volunteers: implications for theories of executive functioning and cognitive aging. Cambridge neuropsychological test automated battery. J Int Neuropsychol Soc. 1998; 4:474–90. 10.1017/s1355617798455073 9745237

[28] Corti EJ, Johnson AR, Riddle H, Gasson N, Kane R, Loftus AM. The relationship between executive function and fine motor control in young and older adults. Hum Mov Sci. 2017; 51:41–50. 10.1016/j.humov.2016.11.001 27842230

[29] Fowler KS, Saling MM, Conway EL, Semple JM, Louis WJ. Computerized neuropsychological tests in the early detection of dementia: prospective findings. J Int Neuropsychol Soc. 1997; 3:139–46. 9126855

[30] Gonçalves MM, Pinho MS, Simões MR. Test-retest reliability analysis of the cambridge neuropsychological automated tests for the assessment of dementia in older people living in retirement homes. Appl Neuropsychol Adult. 2016; 23:251–263. 10.1080/23279095.2015.1053889 26574661

[31] Barnett JH, Blackwell AD, Sahakian BJ, Robbins TW. The paired associates learning (PAL) test: 30 years of CANTAB translational neuroscience from laboratory to bedside in dementia research. Curr Top Behav Neurosci. 2016; 28:449–74. 10.1007/7854_2015_5001 27646012

[32] Lee A, Tan M, Qiu A. Distinct aging effects on functional networks in good and poor cognitive performers. Front Aging Neurosci. 2016; 8:215. 10.3389/fnagi.2016.00215 27667972 PMC5016512

[33] Gruszka A, Hampshire A, Barker RA, Owen AM. Normal aging and parkinson’s disease are associated with the functional decline of distinct frontal-striatal circuits. Cortex. 2017; 93:178–92. 10.1016/j.cortex.2017.05.020 28667892 PMC5542042

[34] Litvan I, Goldman JG, Tröster AI, Schmand BA, Weintraub D, Petersen RC, Mollenhauer B, Adler CH, Marder K, Williams-Gray CH, Aarsland D, Kulisevsky J, Rodriguez-Oroz MC, et al. Diagnostic criteria for mild cognitive impairment in parkinson’s disease: movement disorder society task force guidelines. Mov Disord. 2012; 27:349–56. 10.1002/mds.24893 22275317 PMC3641655

[35] Graham JM, Sagar HJ. A data-driven approach to the study of heterogeneity in idiopathic parkinson’s disease: identification of three distinct subtypes. Mov Disord. 1999; 14:10–20. 10.1002/1531-8257(199901)14:1<10::aid-mds1005>3.0.co;2-4 9918339

[36] Williams-Gray CH, Goris A, Foltynie T, Brown J, Maranian M, Walton A, Compston DA, Sawcer SJ, Barker RA. Prevalence of the LRRK2 G2019S mutation in a UK community based idiopathic parkinson's disease cohort. J Neurol Neurosurg Psychiatry. 2006; 77:665–67. 10.1136/jnnp.2005.085019 16614029 PMC2117467

[37] Jaillard A, Naegele B, Trabucco-Miguel S, LeBas JF, Hommel M. Hidden dysfunctioning in subacute stroke. Stroke. 2009; 40:2473–79. 10.1161/STROKEAHA.108.541144 19461036

[38] Hommel M, Miguel ST, Naegele B, Gonnet N, Jaillard A. Cognitive determinants of social functioning after a first ever mild to moderate stroke at vocational age. J Neurol Neurosurg Psychiatry. 2009; 80:876–80. 10.1136/jnnp.2008.169672 19357128

[39] Wright MJ Jr, Vandewater SA, Parsons LH, Taffe MA. Δ(9)Tetrahydrocannabinol impairs reversal learning but not extra-dimensional shifts in rhesus macaques. Neuroscience. 2013; 235:51–58. 10.1016/j.neuroscience.2013.01.018 23333671 PMC3595391

[40] Weed MR, Bryant R, Perry S. Cognitive development in macaques: attentional set-shifting in juvenile and adult rhesus monkeys. Neuroscience. 2008; 157:22–28. 10.1016/j.neuroscience.2008.08.047 18805462

[41] Rodriguez JS, Zürcher NR, Bartlett TQ, Nathanielsz PW, Nijland MJ. CANTAB delayed matching to sample task performance in juvenile baboons. J Neurosci Methods. 2011; 196:258–63. 10.1016/j.jneumeth.2011.01.012 21276821 PMC3065780

[42] Zürcher NR, Rodriguez JS, Jenkins SL, Keenan K, Bartlett TQ, McDonald TJ, Nathanielsz PW, Nijland MJ. Performance of juvenile baboons on neuropsychological tests assessing associative learning, motivation and attention. J Neurosci Methods. 2010; 188:219–25. 10.1016/j.jneumeth.2010.02.011 20170676 PMC3409834

[43] Spinelli S, Pennanen L, Dettling AC, Feldon J, Higgins GA, Pryce CR. Performance of the marmoset monkey on computerized tasks of attention and working memory. Brain Res Cogn Brain Res. 2004; 19:123–37. 10.1016/j.cogbrainres.2003.11.007 15019709

[44] Spinelli S, Ballard T, Feldon J, Higgins GA, Pryce CR. Enhancing effects of nicotine and impairing effects of scopolamine on distinct aspects of performance in computerized attention and working memory tasks in marmoset monkeys. Neuropharmacology. 2006; 51:238–50. 10.1016/j.neuropharm.2006.03.012 16678864

[45] McEntire CR, Choudhury GR, Torres A, Steinberg GK, Redmond DE Jr, Daadi MM. Impaired arm function and finger dexterity in a nonhuman primate model of stroke: motor and cognitive assessments. Stroke. 2016; 47:1109–16. 10.1161/STROKEAHA.115.012506 26956259

[46] Choudhury GR, Daadi MM. Charting the onset of parkinson-like motor and non-motor symptoms in nonhuman primate model of parkinson’s disease. PLoS One. 2018; 13:e0202770. 10.1371/journal.pone.0202770 30138454 PMC6107255

[47] Kohama SG, Rosene DL, Sherman LS. Age-related changes in human and non-human primate white matter: from myelination disturbances to cognitive decline. Age (Dordr). 2012; 34:1093–110. 10.1007/s11357-011-9357-7 22203458 PMC3448998

[48] Verdier JM, Acquatella I, Lautier C, Devau G, Trouche S, Lasbleiz C, Mestre-Francés N. Lessons from the analysis of nonhuman primates for understanding human aging and neurodegenerative diseases. Front Neurosci. 2015; 9:64. 10.3389/fnins.2015.00064 25788873 PMC4349082

[49] Mitchell SJ, Scheibye-Knudsen M, Longo DL, de Cabo R. Animal models of aging research: implications for human aging and age-related diseases. Annu Rev Anim Biosci. 2015; 3:283–303. 10.1146/annurev-animal-022114-110829 25689319

[50] Peleg S, Sananbenesi F, Zovoilis A, Burkhardt S, Bahari-Javan S, Agis-Balboa RC, Cota P, Wittnam JL, Gogol-Doering A, Opitz L, Salinas-Riester G, Dettenhofer M, Kang H, et al. Altered histone acetylation is associated with age-dependent memory impairment in mice. Science. 2010; 328:753–56. 10.1126/science.1186088 20448184

[51] Chen G, Chen KS, Knox J, Inglis J, Bernard A, Martin SJ, Justice A, McConlogue L, Games D, Freedman SB, Morris RG. A learning deficit related to age and beta-amyloid plaques in a mouse model of alzheimer’s disease. Nature. 2000; 408:975–979. 10.1038/35050103 11140684

[52] Bachevalier J, Landis LS, Walker LC, Brickson M, Mishkin M, Price DL, Cork LC. Aged monkeys exhibit behavioral deficits indicative of widespread cerebral dysfunction. Neurobiol Aging. 1991; 12:99–111. 10.1016/0197-4580(91)90048-o 2052134

[53] Spreng RN, Wojtowicz M, Grady CL. Reliable differences in brain activity between young and old adults: a quantitative meta-analysis across multiple cognitive domains. Neurosci Biobehav Rev. 2010; 34:1178–94. 10.1016/j.neubiorev.2010.01.009 20109489

[54] Samanez-Larkin GR, Knutson B. Decision making in the ageing brain: changes in affective and motivational circuits. Nat Rev Neurosci. 2015; 16:278–89. 10.1038/nrn3917 25873038 PMC5645075

[55] Picq JL, Aujard F, Volk A, Dhenain M. Age-related cerebral atrophy in nonhuman primates predicts cognitive impairments. Neurobiol Aging. 2012; 33:1096–109. 10.1016/j.neurobiolaging.2010.09.009 20970891 PMC3381737

[56] Murman DL. The impact of age on cognition. Semin Hear. 2015; 36:111–21. 10.1055/s-0035-1555115 27516712 PMC4906299

[57] Weed MR, Taffe MA, Polis I, Roberts AC, Robbins TW, Koob GF, Bloom FE, Gold LH. Performance norms for a rhesus monkey neuropsychological testing battery: acquisition and long-term performance. Brain Res Cogn Brain Res. 1999; 8:185–201. 10.1016/s0926-6410(99)00020-8 10556598

[58] Singer T, Lindenberger U, Baltes PB. Plasticity of memory for new learning in very old age: a story of major loss? Psychol Aging. 2003; 18:306–17. 10.1037/0882-7974.18.2.306 12825778

[59] Samanez-Larkin GR, Kuhnen CM, Yoo DJ, Knutson B. Variability in nucleus accumbens activity mediates age-related suboptimal financial risk taking. J Neurosci. 2010; 30:1426–34. 10.1523/JNEUROSCI.4902-09.2010 20107069 PMC2821055

[60] St Onge JR, Ahn S, Phillips AG, Floresco SB. Dynamic fluctuations in dopamine efflux in the prefrontal cortex and nucleus accumbens during risk-based decision making. J Neurosci. 2012; 32:16880–91. 10.1523/JNEUROSCI.3807-12.2012 23175840 PMC6621758

[61] de Boer L, Axelsson J, Riklund K, Nyberg L, Dayan P, Bäckman L, Guitart-Masip M. Attenuation of dopamine-modulated prefrontal value signals underlies probabilistic reward learning deficits in old age. Elife. 2017; 6:e26424. 10.7554/eLife.26424 28870286 PMC5593512

[62] Eppinger B, Heekeren HR, Li SC. Age-related prefrontal impairments implicate deficient prediction of future reward in older adults. Neurobiol Aging. 2015; 36:2380–90. 10.1016/j.neurobiolaging.2015.04.010 26004018

[63] Baron A, Derenne A. Progressive-ratio schedules: effects of later schedule requirements on earlier performances. J Exp Anal Behav. 2000; 73:291–304. 10.1901/jeab.2000.73-291 10866353 PMC1284778

[64] Schwartz N, Temkin P, Jurado S, Lim BK, Heifets BD, Polepalli JS, Malenka RC. Chronic pain. Decreased motivation during chronic pain requires long-term depression in the nucleus accumbens. Science. 2014; 345:535–42. 10.1126/science.1253994 25082697 PMC4219555

[65] Chelonis JJ, Gravelin CR, Paule MG. Assessing motivation in children using a progressive ratio task. Behav Processes. 2011; 87:203–09. 10.1016/j.beproc.2011.03.008 21507343

[66] Schultz W, Apicella P, Ljungberg T. Responses of monkey dopamine neurons to reward and conditioned stimuli during successive steps of learning a delayed response task. J Neurosci. 1993; 13:900–13. 10.1523/JNEUROSCI.13-03-00900.1993 8441015 PMC6576600

[67] Lammel S, Lim BK, Ran C, Huang KW, Betley MJ, Tye KM, Deisseroth K, Malenka RC. Input-specific control of reward and aversion in the ventral tegmental area. Nature. 2012; 491:212–17. 10.1038/nature11527 23064228 PMC3493743

[68] Murty VP, Shah H, Montez D, Foran W, Calabro F, Luna B. Age-related trajectories of functional coupling between the VTA and nucleus accumbens depend on motivational state. J Neurosci. 2018; 38:7420–27. 10.1523/JNEUROSCI.3508-17.2018 30030394 PMC6104300

[69] Palfi S, Ferrante RJ, Brouillet E, Beal MF, Dolan R, Guyot MC, Peschanski M, Hantraye P. Chronic 3-nitropropionic acid treatment in baboons replicates the cognitive and motor deficits of huntington's disease. J Neurosci. 1996; 16:3019–25. 10.1523/JNEUROSCI.16-09-03019.1996 8622131 PMC6579050

[70] Schneider JS, Roeltgen DP. Delayed matching-to-sample, object retrieval, and discrimination reversal deficits in chronic low dose MPTP-treated monkeys. Brain Res. 1993; 615:351–54. 10.1016/0006-8993(93)90049-s 8364742

[71] Diamond A. Developmental time course in human infants and infant monkeys, and the neural bases of, inhibitory control in reaching. Ann N Y Acad Sci. 1990; 608:637–69. 10.1111/j.1749-6632.1990.tb48913.x 2075965

[72] Taylor JR, Elsworth JD, Roth RH, Sladek JR Jr, Redmond DE Jr. Cognitive and motor deficits in the acquisition of an object retrieval/detour task in MPTP-treated monkeys. Brain. 1990; 113:617–37. 10.1093/brain/113.3.617 2364263

[73] Cabré R, Naudí A, Dominguez-Gonzalez M, Ayala V, Jové M, Mota-Martorell N, Piñol-Ripoll G, Gil-Villar MP, Rué M, Portero-Otín M, Ferrer I, Pamplona R. Sixty years old is the breakpoint of human frontal cortex aging. Free Radic Biol Med. 2017; 103:14–22. 10.1016/j.freeradbiomed.2016.12.010 27979658

[74] Havill LM, Mahaney MC, Cox LA, Morin PA, Joslyn G, Rogers J. A quantitative trait locus for normal variation in forearm bone mineral density in pedigreed baboons maps to the ortholog of human chromosome 11q. J Clin Endocrinol Metab. 2005; 90:3638–45. 10.1210/jc.2004-1618 15755864

[75] Ndung'u M, Hartig W, Wegner F, Mwenda JM, Low RW, Akinyemi RO, Kalaria RN. Cerebral amyloid beta(42) deposits and microvascular pathology in ageing baboons. Neuropathol Appl Neurobiol. 2012; 38:487–99. 10.1111/j.1365-2990.2011.01246.x 22126319

[76] Schultz C, Hubbard GB, Rub U, Braak E, Braak H. Age-related progression of tau pathology in brains of baboons. Neurobiol Aging. 2000; 21:905–12. 10.1016/s0197-4580(00)00176-7 11124441

